# Failure characteristics and conditions of rock-coal combination structure with weak layer under dynamic and static stresses

**DOI:** 10.1038/s41598-023-39427-5

**Published:** 2023-07-31

**Authors:** Yufeng Fan, Xiaochun Xiao, Jun Xu, Xin Ding, Aiwen Wang, Beifang Wang, Yun Lei

**Affiliations:** 1 School of Mechanics and Engineering, Liaoning Technical University, Fuxin, Liaoning 123000 China; 2 Liaoning Key Laboratory of Mining Environment and Disaster Mechanics, Liaoning Technical University, Fuxin, Liaoning 123000 China; 3 Institute of Disaster Rock Mechanics, Liaoning University, Shenyang, Liaoning 110000 China; 4 School of Mines, Liaoning Technical University, Fuxin, Liaoning 123000 China; 5 Shenyang Research Institute, China Coal Technology and Engineering Group Corp, Fushun, 113122 China; 6 State Key Laboratory of Coal Mine Safety Technology, Fushun, 113122 China

**Keywords:** Fossil fuels, Coal

## Abstract

To comprehensively understand the mechanical response of a rock-coal combination structure containing a weak layer, a series of laboratory static loading and impact loading experiments were conducted. The results showed that under static load, the sliding process of the rock coal structure was relatively slow, and fragments can be observed. Under the action of horizontal impact loading, the whole coal stratum slipped out rapidly, and the process lasted only 0.05 s. Under the horizontal and axial impact loads, the coal stratum remained stable first, and then it slipped out as a whole under the action of static load. Additionally, a sliding instability criterion of a rock coal structure containing a weak layer was established based on theoretical analysis. The key parameter P value was checked through a numerical simulation experiment. It was found that the value was linearly related to the mechanical properties of the weak layer and overburden stress, and the experimental results coincided with the theoretical results. Finally, the relationship between sliding rockburst and strain rockburst was discussed, and these results can provide an important scientific basis for the prevention and control of dynamic disasters in deep mining.

## Introduction

**Figure 1:**
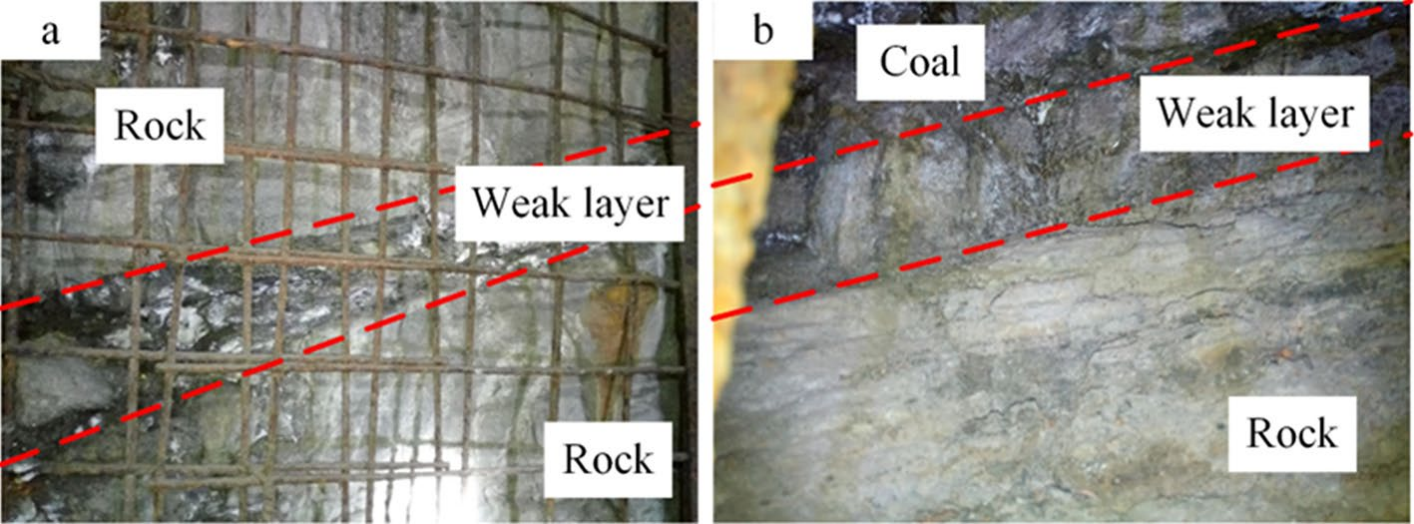
Weak layer in roadway surrounding rock.

In the roadway, the roof rock seam, floor rock seam, and coal pillar constitute a strong nonlinear engineering rock mass. The rock system is the main carrier of energy absorption in the face of static and dynamic load caused by goaf roof fracture or ground stress adjustment. Typically, a weak layer (with poor integrity and properties) usually exists between the rock and coal seam. The mechanical properties of the weak layer often have a significant impact on the stability of the surrounding rock of the roadway^1,2^, as shown in Fig. 1. The long-term stability control of coal pillars is one of the main challenges to ensure roadway safety. Therefore, it is of great significance to clarify the initiation of slip in weak rock-coal combination structures under static and dynamic loads for controlling the surrounding rock of roadway.

According to the International Society of Rock Mechanics (ISRM), extrusion is a large time-dependent deformation that occurs during tunnel construction, and extrusion is associated with creep caused by exceeding the ultimate shear stress^3^. In deep mines, the roadway is generally in the coal seam, subject to high ground stress, high temperature, and high gas pressure, as well as complex geological conditions, maintaining the stability of the roadway surrounding rock is the primary condition to ensure production safety. The deformation and stability of roadway surrounding rock are mainly caused by the extrusion stress, which is generally caused by the subsidence of goaf roof^4–7^. Through physical test, theoretical analysis and numerical simulation, many previous studies focused on the main factors affecting the deformation of surrounding rock and the failure forms^8–14^, and put forward the corresponding empirical methods to predict the tunnel compression conditions^15^. The deformation and damage of surrounding rock in inclined coal seam and adjacent goaf of roadway are mainly considered^16–21^. By combining theoretical analysis, numerical simulation and field measurement, Fang et al.^22^ discussed the deformation characteristics and influencing factors of surrounding rock of roadway. Aiming at the roadway support problem, numerical software such as Flac and UDEC are widely used to determine the deformation and damage of roadway surrounding rock support methods and supporting structures^23,24^. The supporting methods of soft rock roadway and thick top coal roadway are important measures to ensure the stability of roadway surrounding rock^25–30^. These research results showed that the extrusion stress was an important factor affecting the stability of roadway surrounding rock, which provided an important reference for the determination of the critical lateral pressure coefficient of roadway surrounding rock instability in this paper. It also guided the numerical simulation of this study.

In addition to the extrusion caused by the evolution of the stress field of the surrounding rock, the surrounding rock structure of the roadway is also one of the main factors affecting rock burst. Especially, the small coal pillar in goaf roadway is prone to rock burst disaster due to stress concentration or dynamic load disturbance^31,32^. The asymmetry of stress environment of roadway surrounding rock will also lead to uneven distribution of plastic zone of floor^33^. The strength and width of coal pillar will affect the deformation and failure of surrounding rock^34^. Many previous studies analyzed the deformation characteristics of the roadway surrounding rock under different coal pillar widths and stress concentration coefficients and systematically studied the influence of the overburden structure and coal pillar width on the deformation mechanism of the roadway under load during the mining process^35,36^. Wang et al.^37^ used the theoretical analysis method to establish a structural mechanics model of overburden rock along hollow tunneling considering the gangue compression effect and analyzed the synergistic control effect of the "coal wall (column)-gangue" bearing system on the stability of overburden rock. The above research shows that the stress redistribution caused by geological structural factors is an important factor leading to rockburst. Therefore, the structural factors of roadway surrounding rock should be considered emphatically.

This paper considers that the sliding instability of small coal pillar roadway with weak layer is affected by dynamic load disturbance and its own structural factors. When the mechanical properties of the weak layer are deteriorated or destroyed, the broken rock block can weaken the bonding between the rock layer and the coal seam, which may induce slip damage of the coal seam and even lead to slipping rockburst disasters. Therefore, it is necessary to study the mechanical response of rock-coal combination structures with weak layers under overburden stress and dynamic load disturbance. In this study, the judgment method of weak layer fracture was proposed, and the parameters were checked and verified by numerical simulation. Finally, sliding tests of rock coal combination structures were carried out through laboratory experiments under various load conditions, and the variation characteristics of the displacement field were monitored in the process of fracture.

## Slip fracture criterion of a combined coal and rock structure containing a weak layer

### Stress field distribution and dynamic load disturbance form of roadway surrounding rock

**Figure 2:**
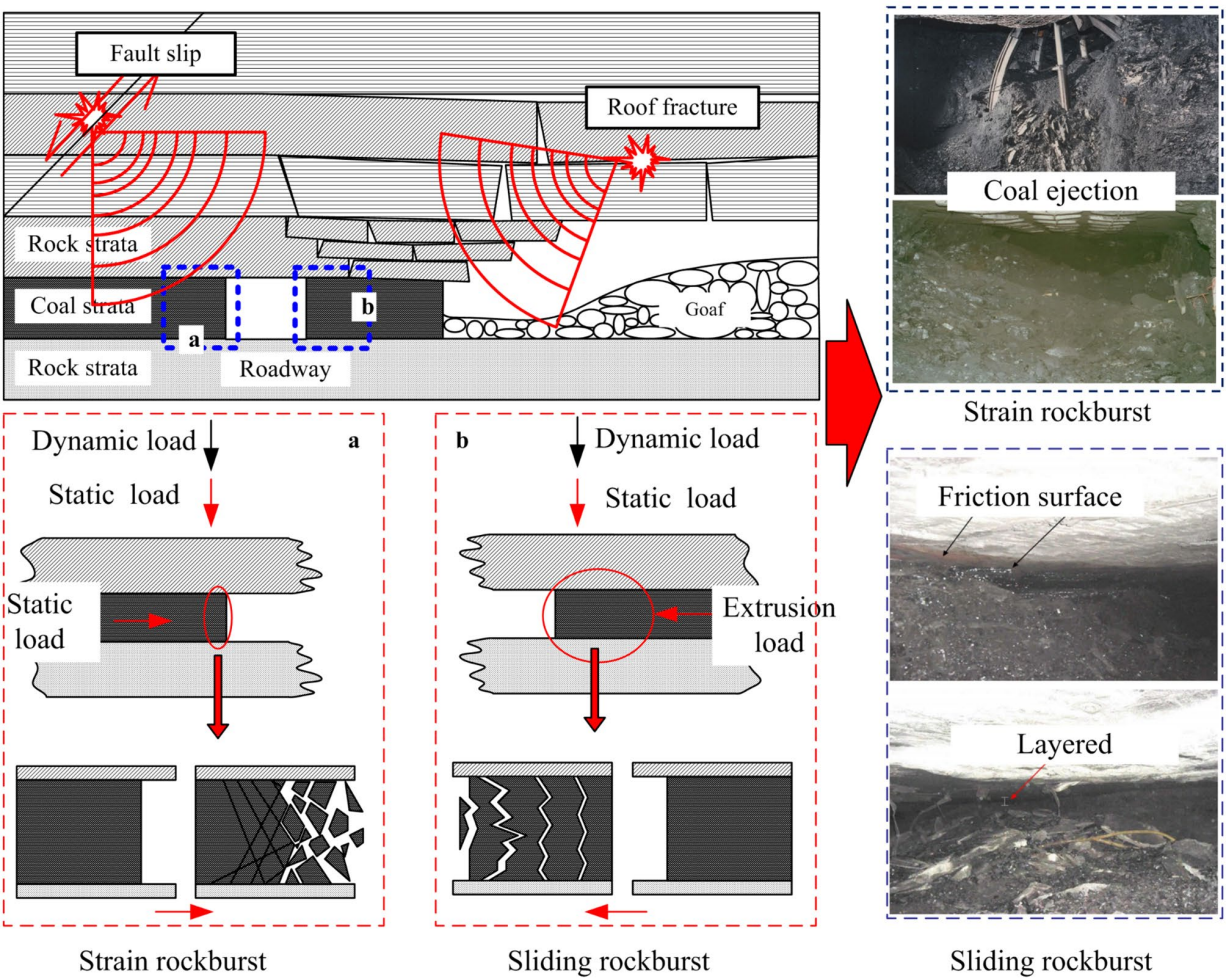
Stress field distribution and dynamic load disturbance form of roadway surrounding rock.

The deep mine is affected by complex geological conditions, in situ stress distribution and mining operation. The stress distribution and evolution mode of the roadway surrounding rock are complex, especially the roof settlement of the goaf, which may lead to the extrusion of coal seam. As shown in Fig. 2, the surrounding rock of a deep roadway has the following characteristics:

1. After roadway excavation, to avoid the near-field coal seam stress concentration caused by roof settlement, drilling pressure relief is generally required to transfer the stress concentration area to the deep part of the coal seam.
2. The subsidence of the goaf roof leads to the extrusion of the coal pillar. After the stress concentration area of the near-field surrounding rock of the roadway is transferred to the deep, the lateral pressure coefficient of the near-field surrounding rock will be higher than the initial value.
3. There is often a layer of rock (weak layer) with poor integrity between the seam and the coal seam. When the lateral pressure coefficient increases, it is easy to slide and destroy along the weak layer, resulting in the overall inclination of the coal seam.
4. The dynamic disturbance loading caused by far-field fault slip or dislocation will act on the vertical and horizontal directions of the rock-coal combination structure at the same or different times, as shown in Fig. 2a.
5. The dynamic disturbance loading caused by roof blasting activity or roof fracture also acts on the rock-coal combination structure in the form of impact disturbance, as shown in Fig. 2b.

In conclusion, with the adjustment of the in situ stress of the surrounding rock, it is possible that the vertical stress in the ground could decrease while the horizontal stress could increase. The weak layers between rock seam may cause the sliding rock burst to be readily induced under a dynamic load disturbance, especially on the coal pillar side near the goaf. The side coal pillar in the goaf shown in Fig. 2b is taken as the main research object.

### Sliding instability criterion of roadway surrounding rock

Considering the influence of horizontal ground stress on the slip fracture of weak layers, the vertical ground stress direction is defined as the z-axis, and the direction along the roadway strike is defined as the x-axis. The direction of the coal seam perpendicular to the roadway is defined as the y-axis.

**Figure 3:**
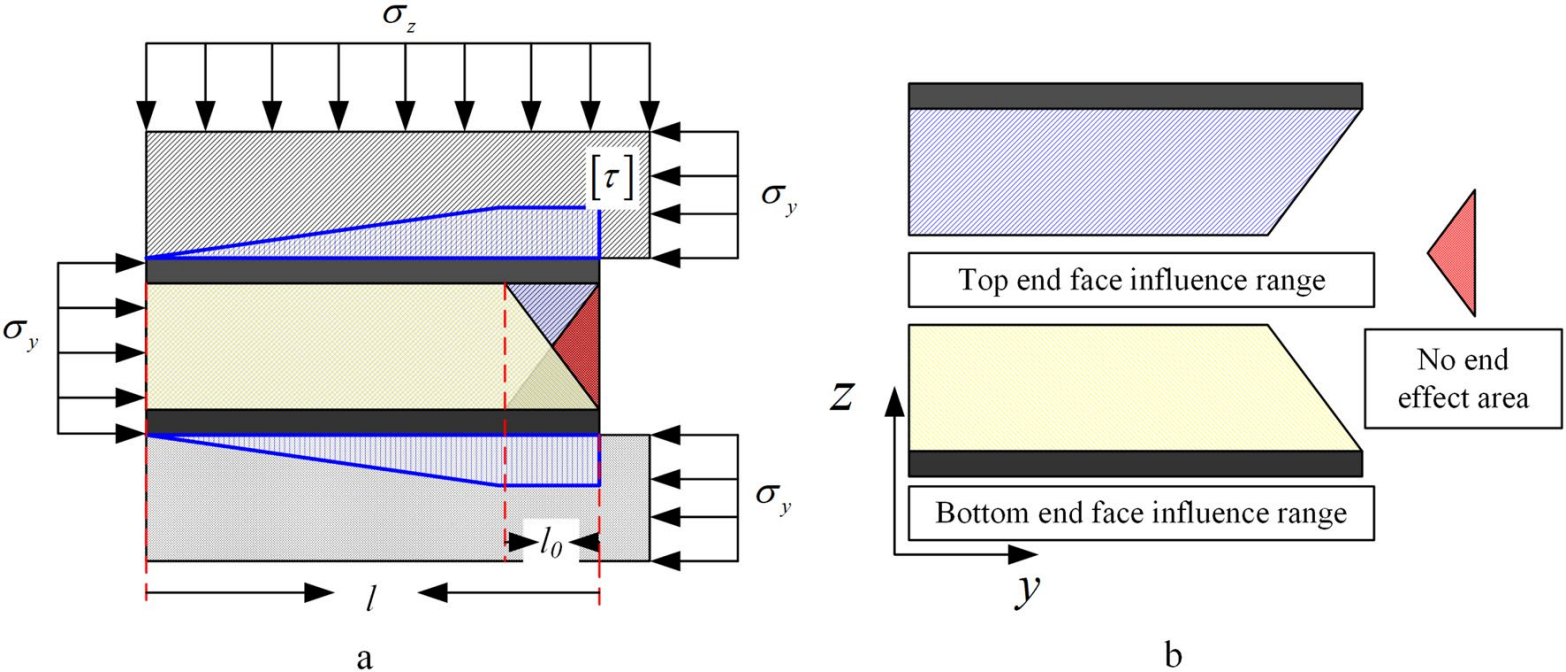
Combined structural mechanics model. (**a**) Stress distribution of the surrounding rock. (**b**) Near-field alleywall shear stress distribution.

As shown in Fig. 3a, the combined structure formed by the coal seam and the weak layer is affected by the ground stress $\sigma_{y}$ in the horizontal direction. The weak layer provides shear stress to maintain the static balance of the structure. The lateral pressure ratio coefficient $K_{\lambda }$ can be expressed as

1 $$K_{\lambda } = \frac{{\sigma_{y} }}{{\sigma_{z} }}$$

The shear stress satisfies the Mohr‒Coulomb criterion

2 $$\left[ \tau \right] = C + \sigma_{z} \tan \left( \phi \right)$$

where $\left[ \tau \right]$ is the maximum shear stress, $C$ is the cohesion of the weak layer, and $\phi$ is the internal friction angle. The damage of the weak layer does not occur when the horizontal load is less than the maximum horizontal shear, which can be expressed as

3 $$F_{y} = \int_{0}^{{h_{c} }} {K_{\lambda } \sigma_{z} } dz < 2F_{s} = 2\int_{0}^{l} {\left[ \tau \right]dy}$$

where $F_{y}$ is the horizontal load, $F_{s}$ is the horizontal shear provided by the weak layer, $h_{c}$ is the thickness of the coal seam, and $l$ is the depth of the coal seam. The relationship between horizontal ground stress and shear stress can be expressed as

4 $$\left[ \tau \right] \ge \frac{{K_{\lambda } \sigma_{z} h_{c} }}{2l}$$

According to Eqs. (2) and (4), the relationship between the lateral pressure ratio coefficient $K_{\lambda }$ and the stress can be obtained

5 $$K_{\lambda } \le \frac{{2l\left[ {C + \sigma_{z} \tan \left( \phi \right)} \right]}}{{\sigma_{z} h_{c} }}$$

Equation (5) reflects the range of lateral pressure ratio coefficients that keep the weak layer stable, and the weak layer is stable when the lateral pressure ratio coefficient satisfies equation. The structural body composed of a weak layer and coal satisfies the static equilibrium of the external load and weak layer shear force on the engineering scale. However, since the coal seam have a certain height, generally at 3–6 m, the shear force varies at different distances from the weak layer. To analyze the stress state in the near field of the roadway, the combined model can be simplified, as shown in Fig. 3b.

The shear stress in the coal seam was superimposed by the shear force provided by the upper and lower weak layers, and the vertical ground stress was simplified to a uniform load. It can be assumed that there exists a point at the near field of the roadway at a distance of *l*_*0*_ from the roadway boundary where the shear stress was 0. First, the horizontal stress at a distance *l*_*0*_ location is determined, and according to the superposition of the upper and lower end faces, it can be written as

6 $$\sigma_{y} h_{c} - 2\int_{0}^{h} {\frac{\partial \left[ \tau \right]}{{\partial z}}\left( {l - \frac{5}{4}l_{0} } \right)} dz = \sigma_{y0} h_{c}$$

where *l*_*0*_ is the distance from the roadway boundary and $\sigma_{y0}$ is the horizontal stress at the location, which can be written as

7 $$\sigma_{y0} = \frac{{5\left[ \tau \right]l_{0} }}{{2h_{c} }}$$

Similarly, to satisfy the weak layer stability at the near field of the roadway, the shear stress provided by the weak layer can be written as

8 $$\tau_{0} = \frac{5\left[ \tau \right]}{4}$$

Additionally, the condition for the stability of the weak layer in the near field of the roadway can be written as

9 $$K_{{\lambda_{0} }} \le \frac{{8l\left[ {C + \sigma_{z} \tan \left( \phi \right)} \right]}}{{5\sigma_{z} h_{c} }}$$

where $K_{{\lambda_{0} }}$ in Eq. (9) is the side pressure ratio coefficient in the near field of the roadway. Comparing Eq. (5) and Eq. (9), when the side pressure ratio coefficient meets the stability of the weak layer in the far field, the weak layer in the near field of the roadway may also be damaged. It should be noted that the side pressure ratio coefficient must meet both Eqs. (5) and (9) to ensure the stability of the weak layer in the roadway, that is, to meet Eq. (10).

10 $$K_{\lambda }^{W} \le \frac{{8l\left[ {C + \sigma_{z} \tan \left( \phi \right)} \right]}}{{5\sigma_{z} h_{c} }}$$

where $K_{\lambda }^{W}$ is the weak layer slip lateral pressure ratio coefficient. In addition, to calculate the relationship between *l*_*0*_ and the depth $l$ of the roadway, the shear stress $\tau_{0}$ should be equal to $\left[ \tau \right]$ at the near field of the roadway, and the horizontal stress $\sigma_{y0} h_{c}$ should be equal to $2\left[ \tau \right]l_{0}$ at the location *l*_*0*_. According to Eq. (10), the horizontal stress can be obtained, as shown in Eq. (11).

11 $$K_{\lambda }^{W} \sigma_{z} h_{c} { = }\frac{4}{5}\left( {2\left[ \tau \right]l} \right)$$

According to the static equilibrium, the relationship of the horizontal direction shear force and load can be expressed as Eq. (12).

12 $$K_{\lambda }^{W} \sigma_{z} h_{c} = \frac{8l\left[ \tau \right]}{5} = \left( {l - l_{0} } \right)\left[ \tau \right] + 2l_{0} \left[ \tau \right]$$

Relationship between the near-field depth *l*_*0*_ and the far-field depth $l$ of the alleyway.

13 $$l_{0} = \frac{3}{5}l$$

According to Eqs. (10) and (13), it was easy to find that when the shear strength of the weak layer increases, the maximum lateral pressure ratio coefficient allowed also increases. When the shear strength is specific, the change in the lateral pressure ratio coefficient can increase the influence range of the near- and far-field depths of the roadway. In the far field of the roadway, the weak layer is more stable due to the minor shear stress, so it is necessary to determine the relationship between the far-field depth $l$ and the coal seam thickness $h_{c}$ in the actual determination, and the determination method will be analyzed and discussed in detail in section "Numerical simulation and calibration experiment of the criterion".

When shear damage occurs somewhere in the weak layer, the cohesion within the weak layer disappears, and the weak layer is subject to vertical ground stresses providing only frictional forces.

14 $$f = \mu N = \tan \left( \phi \right)\int_{0}^{l} {\sigma_{z} } dy$$

where $f$ is the sliding friction, $\mu$ is the friction coefficient, $N$ is the vertical load, and the weak layer shear force is far from maintaining the stability of the weak layer. Under the action of horizontal load, weak layer damage gradually evolves. The evolution rate gradually accelerates, so to prevent roadway slip impact hazards, the side pressure ratio should meet the side pressure ratio $K_{\lambda }^{W}$.

After the rock surrounding the tunnel is depressurized by drilling, the stress concentration area of the in situ stress shifts to greater depths. When the local stress exceeds the strength of the coal seam, the coal seam will be damaged. The in situ stress at fracture can be expressed as:

15 $$\sigma_{z} \ge C_{c} + \sigma_{z} \tan \phi_{c}$$

In Eq. (15), $C_{c}$ and $\tan \phi_{c}$ are the cohesion and friction angle of the coal seam, respectively, so the critical in situ stress $K_{{\sigma_{z} }}$ can be expressed as Eq. (16).

16 $$K_{{\sigma_{z} }} \le \frac{{C_{c} }}{{1 - \tan \phi_{c} }}$$

## Numerical simulation and calibration experiment of the criterion

According to the above analysis of the lateral pressure ratio, mechanical properties of the weak layer and mechanical properties of coal, the theoretical derivation for the slipping fracture judgment of the coal combined structure containing a weak layer, some parameters were still not calibrated, such as the depth of coal seam at the far-field of the roadway $l$. This section will correct and calibrate the lateral pressure ratio coefficient $K_{\lambda }^{W}$ of slip of the weak layer and the judgment coefficient $K_{{\sigma_{z} }}$ of coal damage by a numerical calculation method.

### Slip damage parameter calibration process

**Figure 4:**
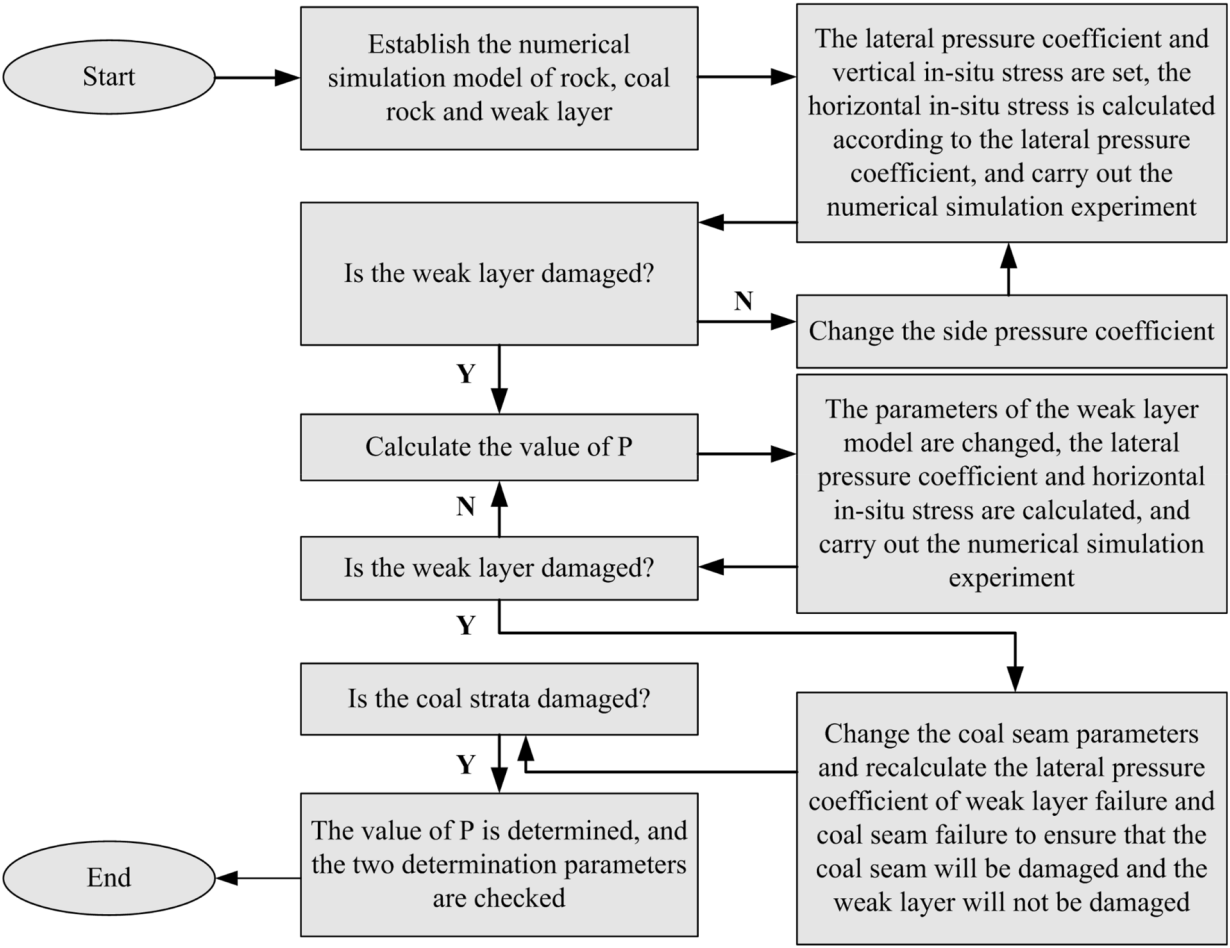
Parameter verification steps.

To calibrate the roadway far-field depth $l$ and the criterion, the steps are shown in Fig. 4.

1. The weak layer slip lateral pressure ratio coefficient $K_{\lambda }^{W}$ and coal damage determination coefficient $K_{\lambda }^{C}$ were calculated based on the value of vertical ground stress $\sigma_{z}$, weak layer mechanical parameters, and coal mechanical parameters.
2. The horizontal stress $\sigma_{y}$ is given, which is equal to $\lambda \sigma_{z}$, and numerical calculations were performed to determine the lateral pressure ratio $\lambda$ when the weak layer breaks precisely, which determined the ratio of the far-field depth to the height of the roadway $P$.
  17 $$P = \frac{l}{{h_{c} }}$$
3. Combined with the experimental results of (2), the mechanical parameters of the weak layer were changed to calculate the weak layer slip lateral pressure ratio $K_{\lambda }^{W}$, the horizontal stress was determined, and the numerical experiment is repeated. If the weak layer happens to be damaged at this stress level, the theoretical analysis of the weak layer slip lateral pressure ratio coefficient is correct.
4. Calculate the coal damage determination coefficient $K_{{\sigma_{z} }}$ based on the experimental results of (2) and change the mechanical parameters of coal to repeat the numerical experiment. The coal seam failed when the parameters were equal to the theoretical critical compressive strength, and the theoretical analysis of the damage determination coefficient of the coal seam was correct.

### Fracture mode of contact between blocks

**Figure 5:**
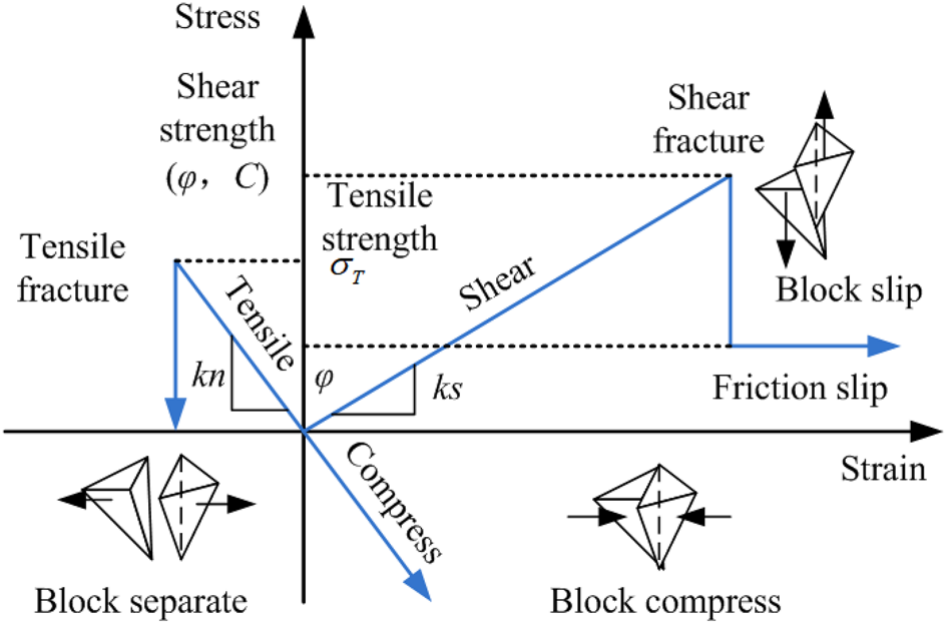
The fracture mode of contact between blocks.

The block discrete element method was used to simulate the sliding instability process of a rock coal composite structure. Taking the rock mass as a general interpretation example, rock blocks (continuum) and geological structural planes (discontinuous features) with different lithologic attributes constituted the most basic elements of the rock mass. Under the action of an external force, rock blocks can behave as continuous medium mechanical behavior, and rock blocks interact through structural planes (discontinuous features). When the stress of structural planes exceeds its bearing limit, rock blocks were shown as realistic fracture phenomena such as mutual shear dislocation or separation. The fracture mode of contact between the blocks is shown in Fig. 5.

### Model establishment

**Figure 6:**
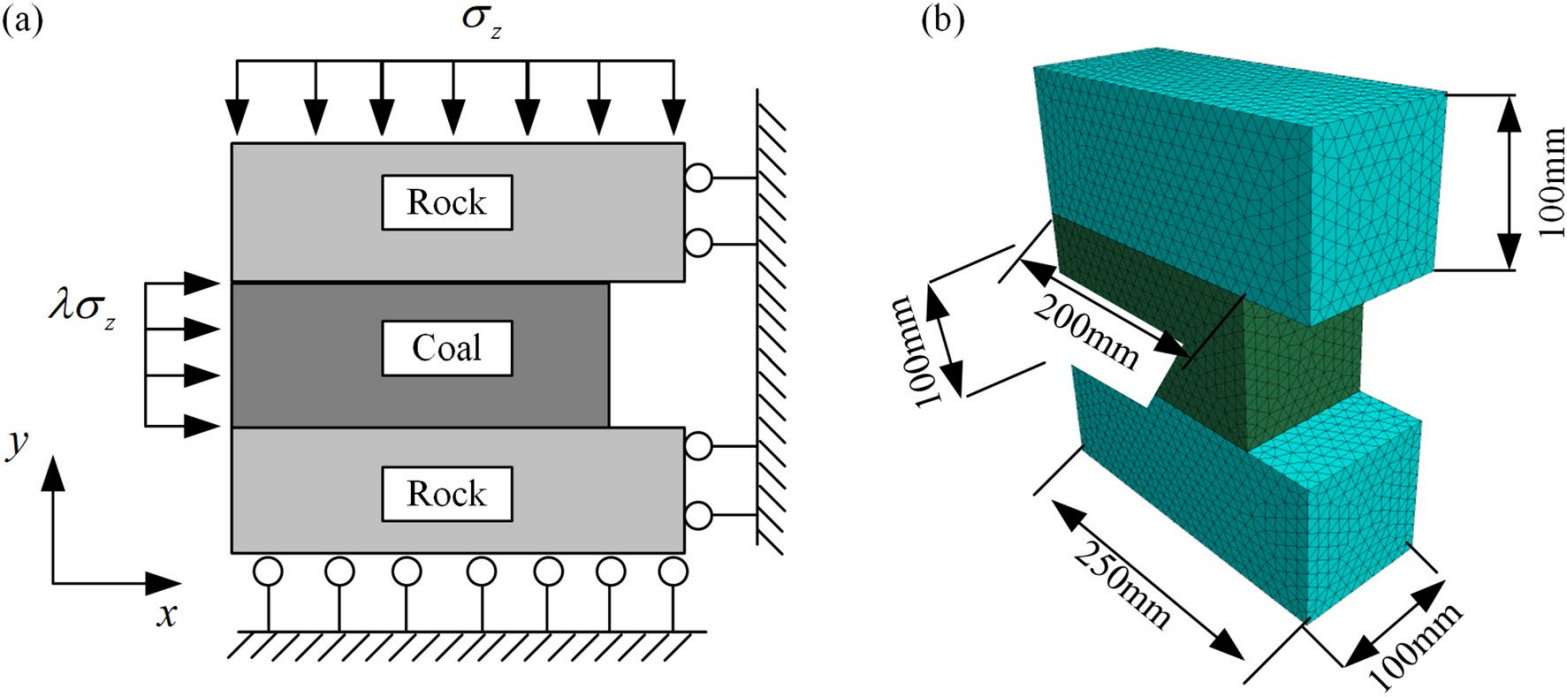
Boundary condition and numerical calculation model.

This study only considered the slip fracture of the weak layer and the fracture of the coal seam, so the rock was defined as completely elastic. The weak layer was represented by the contact between rock and coal. The mechanical properties of the contact surface determine whether the weak layer was damaged. The Mohr‒Coulomb constitutive model was selected for the coal seam. The boundary conditions and geometric model are shown in Fig. 6a. The size of the model is shown in Fig. 6b, and a tetrahedral mesh partition model is adopted with a maximum mesh size of 10mm.

In addition, at the upper boundary, 10 MPa vertical ground stress was applied. Vertical ground stress (16 to 50 MPa) was applied on the other side of the roadway. The model block parameters are shown in Table 1, and the contact parameters are shown in Table 2. It should be noted that the normal stiffness and shear stiffness were used to restrict the maximum allowable overlap between blocks, so they are both taken as 5·10^5^ MPa m.

**Table 1:** Block parameters.

Mechanics properties	Value
Block	
Rock elastic modulus (GPa)	13.5
Rock Poisson's ratio	0.123
Coal elastic modulus (GPa)	5.3
Coal Poisson's ratio	0.2
Coal cohesive strength (MPa)	1.25–5
Coal tension strength (MPa)	0.15–2
Coal friction angle (°)	20–30

**Table 2:** Contact parameters.

Mechanics properties	Value
Connect	
Rock-coal weak layer normal stiffness (MPa m)	500,000
Rock-coal weak layer shear stiffness (MPa m)	500,000
Between weak layer cohesive(residuals) (MPa)	3.2 (0.2)–5 (0.2)
Between weak layer tension (residuals) (MPa)	1.1 (0.2)
Between weak layer friction (residuals) (°)	18 (15)–32 (15)

### Analysis of the calibration results

#### Determination of ***P*** value and $K_{\lambda }^{W}$

**Figure 7:**
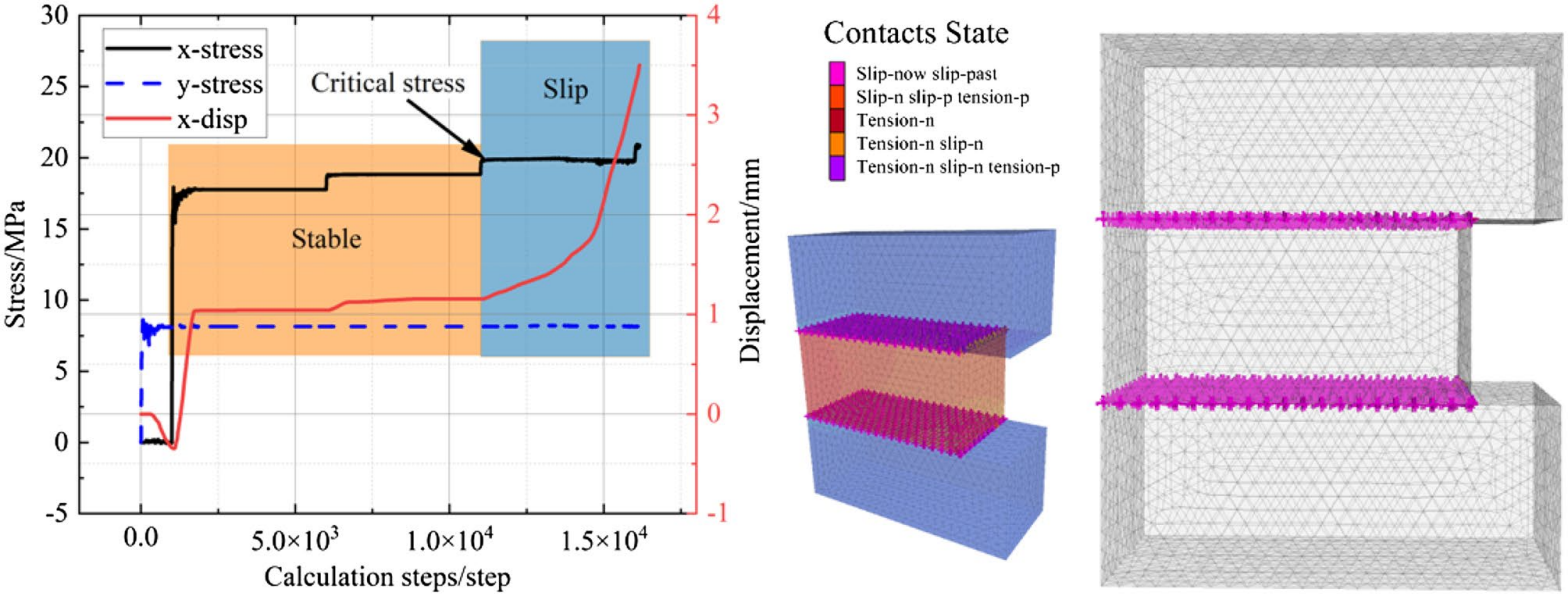
Horizontal displacement curve and weak layer damage.

**Figure 8:**
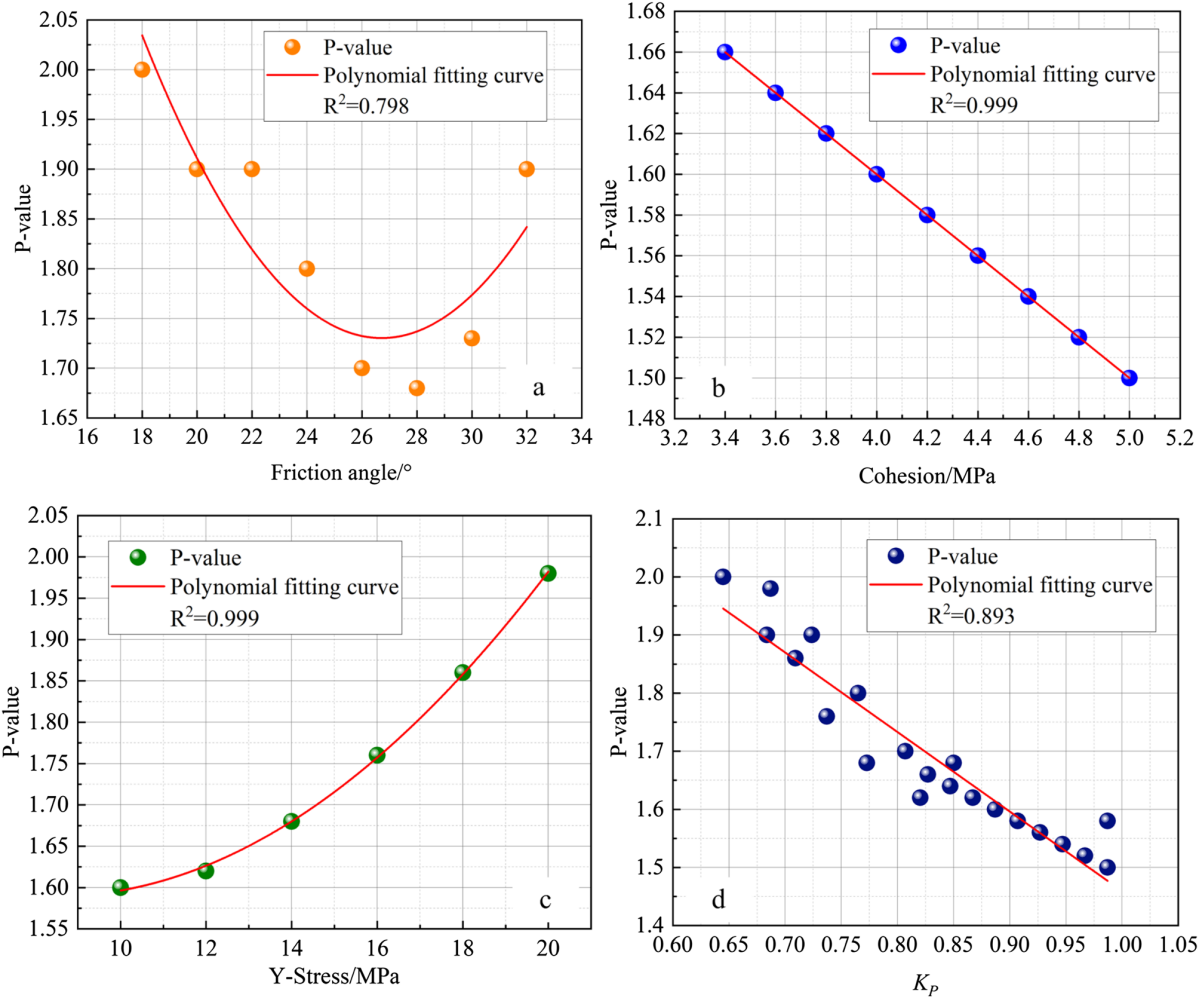
*P value* affected by mechanical parameters of the weak layer. (**a**) Relation of the *P value* and friction angle. (**b**) Relation of the *P value* and cohesion. (**c**) Relation of the *P value* and Y-stress. (**d**) Relation of *P value* and $K_{P}$.

The test monitors the horizontal displacement in the positive y-axis direction at the center of the coal seam. The visualization interface allowed the observation of the weak layer and coal damage and stops the calculation when there was a sudden change in the horizontal deformation. Figure 7 shows the horizontal displacement curve of the coal seam and the weak layer damage. The change in the *P* value is shown in Fig. 8.

Figure 8 shows the influence of the weak layer mechanical properties on the *P* value. There are power function, linear and exponential function relations between the *P* value and friction angle, cohesion and in situ stress, respectively. The ratio $K_{P}$ of the shear strength and in situ stress of the weak layer also has a linear relationship with the *P* value, and the fitting degree was 0.893. $K_{P}$ is:

18 $$K_{P} = \frac{{\left( {C + \sigma_{z} \tan \phi } \right)}}{{\sigma_{z} }}$$

Therefore, the *P* value can be expressed as Eq. (19).

19 $$P = \frac{l}{{h_{c} }} = 2.83 - 1.37\frac{{\left( {C + \sigma_{z} \tan \phi } \right)}}{{\sigma_{z} }}$$

The lateral pressure ratio coefficient $K_{\lambda }^{W}$ can be modified to Eq. (20):

20 $$K_{\lambda }^{W} \le \frac{8}{5}\frac{{\left[ {C + \sigma_{z} \tan \left( \phi \right)} \right]}}{{\sigma_{z} }}P$$

#### Calibration of $K_{{\sigma_{z} }}$

**Figure 9:**
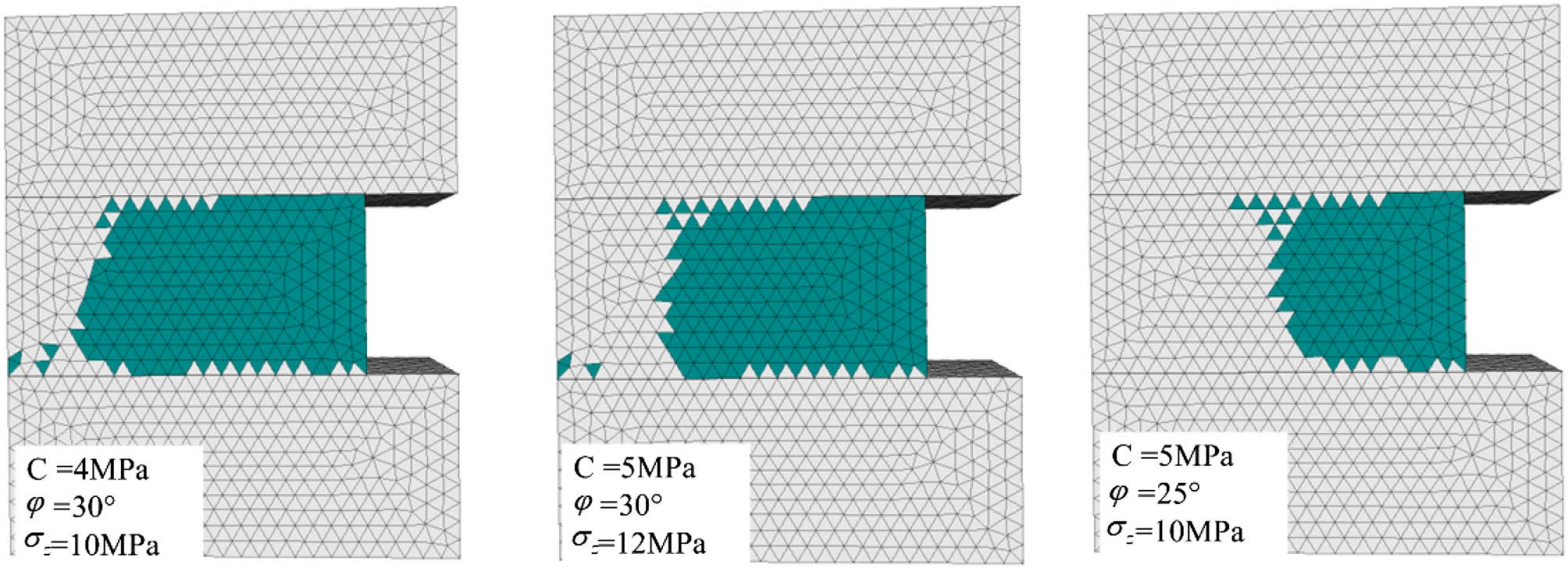
Coal seam fracture characteristics.

Similar to the previous simulation steps, the critical in situ stress of coal seam fracture is explored by changing the mechanical parameters of the coal seam. The parameters and fracture of the coal seam are shown in Fig. 9.

As shown in Fig. 9, when the local stress exceeds the theoretical value, the coal seam will undergo shear fracture. The numerical experimental results showed that the theory proposed above can predict coal damage based on the parameters of the weak layer, coal mechanical properties and stress state. It can also provide a criterion for preventing slip-type impact ground pressure and coal body compression-type impact ground pressure.

## Experimental study on the sliding instability of a rock-coal combination structure with a weak layer under dynamic and static loads

### Specimen preparation and text scheme

**Figure 10:**
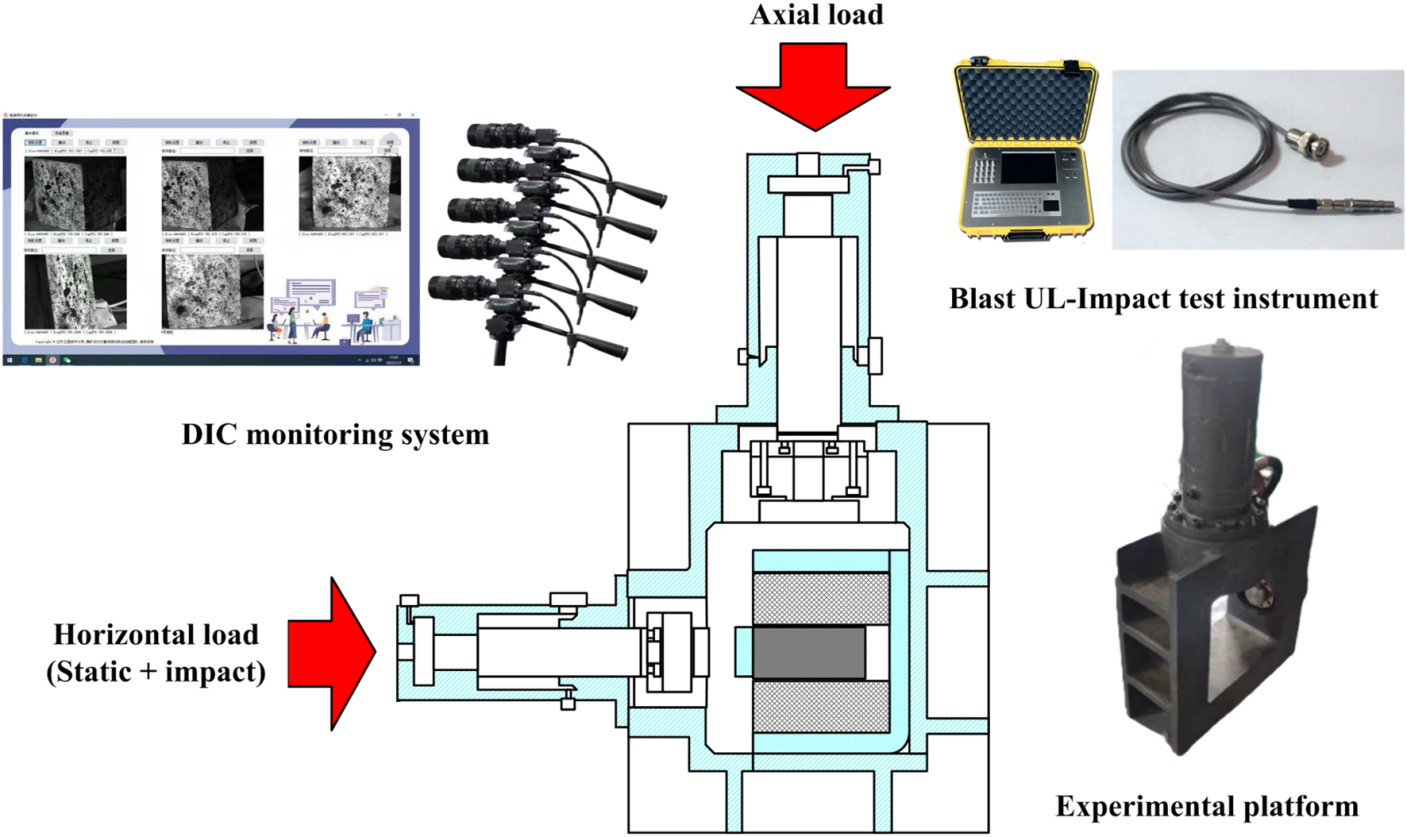
The experimental system.

**Figure 11:**
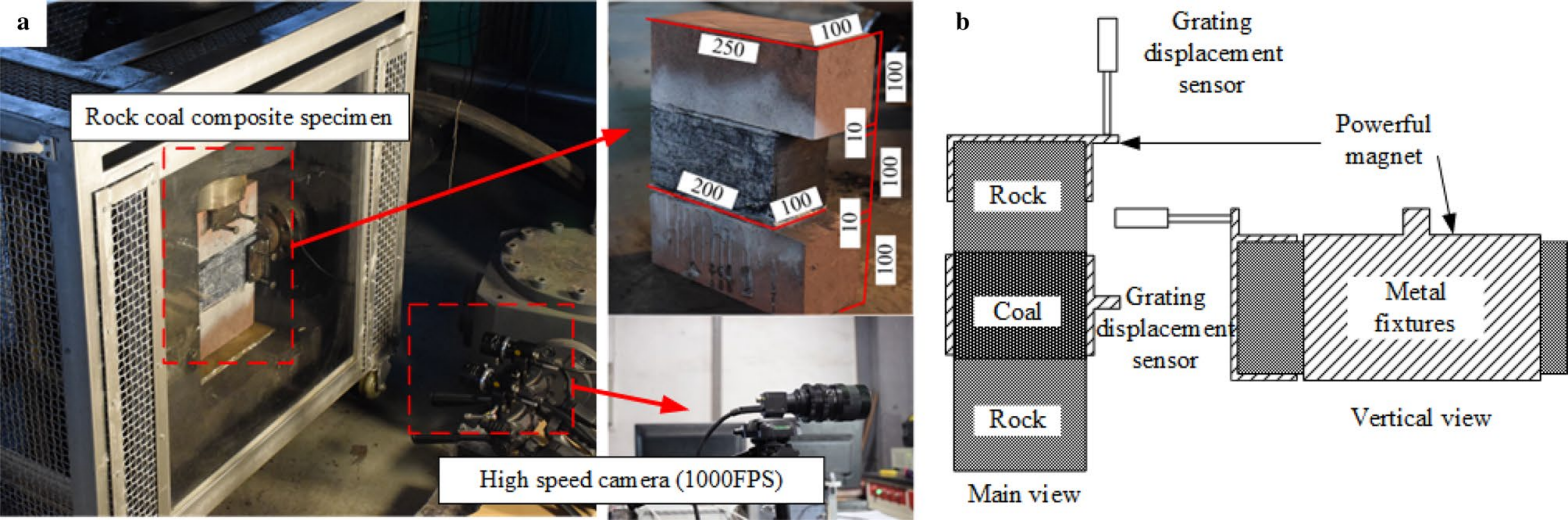
The experimental arrangement.

In order to verify the theoretical criterion in section "Slip fracture criterion of a combined coal and rock structure containing a weak layer" and the numerical calculation results in section "Numerical simulation and calibration experiment of the criterion". The self-developed static and dynamic combined loading test device for rock coal combination structure was used to carry out experimental research. The high-speed camera acquisition system was used to monitor the surface damage process of the specimen and analyze the change characteristics of the surface displacement field. The stress sensor and grating displacement sensor were used to monitor the load change and displacement change in two directions. The experimental system is shown in Fig. 10, and the experimental arrangement is shown in Fig. 11a. Metal fixtures are arranged on the upper surface of the specimen and the coal seam to more accurately monitor the slip of the coal seam and the small deformations of the rock system, as shown in Fig. 11b. The test piece used in the test was a combination of rock and coal with a weak layer, and the preparation method was as follows:

1. The coal was selected from a mining area in Inner Mongolia in China and cut into 100 mm × 200 mm × 100 mm rectangular specimens.
2. Red sandstone was selected and cut into 100 mm × 250 mm × 100 mm rectangular specimens.
3. The weak layer was prepared by mixing quartz sand, gypsum, cement, retarder and water, and the size is 100 mm × 200 mm × 10 mm. The sheet test piece was prepared after curing for 14 days, and the mechanical parameters are measured. The mechanical parameters are shown in Table 3.
4. The rock specimen, coal specimen and weak layer specimen prepared in (1), (2) and (3) were bonded by high-strength epoxy resin in the order of rock weak layer coal rock weak layer rock, and the surface of the combined specimen was sprayed with speckles. The test scheme is shown in Table 4.

**Table 3:** Mechanical parameters of the weak layer.

Mechanics properties	Value
Density (g/cm^3^)	1.59
Cohesive (MPa)	3.2
Poisson's ratio	0.22
Tension strength (MPa)	1.3
Friction angle (°)	13.3

**Table 4:** Test scheme.

Specimen	Loading condition	Horizontal static load/t	Axial static load/t	Horizontal impact/t	Axial impact/t	Fracture characteristics
S-1	Static	3.6	7	–	–	No slip
S-2	Static	7	7	–	–	No slip
S-3	Static	11	7	–	–	No slip
S-4	Static	14	7	–	–	Conical fracture, overall dumping
D-1	Horizontal impact	10	7	2	–	No slip
D-2	Horizontal impact	10	7	5	–	Violent coal seam dumping
DD-1	Double impact	10	7	1	5	No slip
DD-2	Double impact	10	7	2	5	After the impact is applied, it is stable, and then it is poured out as a whole

### Test result

#### Sliding conditions of rock coal structure affected by loading mode

**Figure 12:**
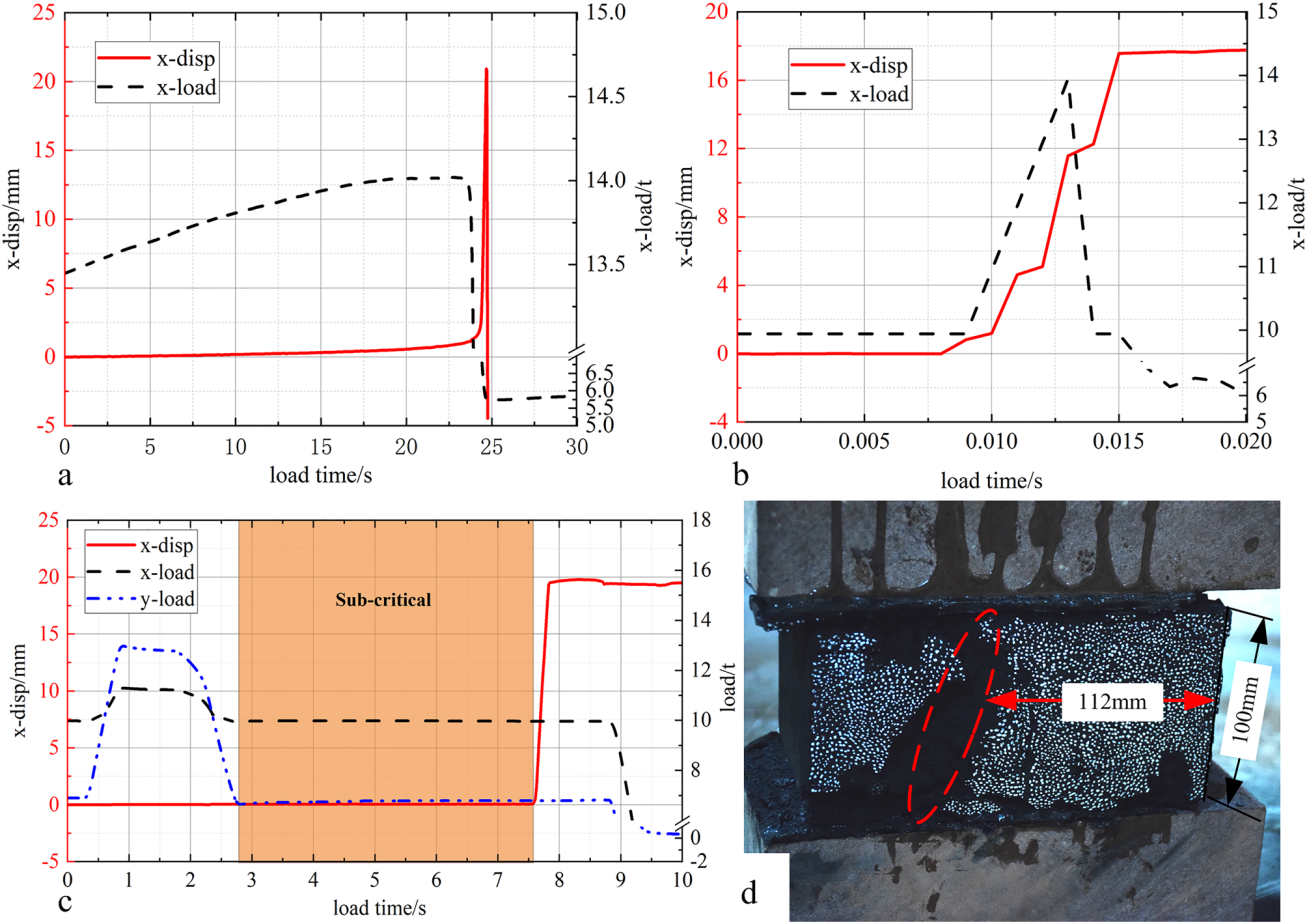
Load and displacement curves in the horizontal direction with time.

First, the sliding instability experiment of a rock coal structure under static load is carried out, and the static load critical condition of structural instability was determined, as shown in Fig. 12a. On this basis, the horizontal impact load was applied, and the horizontal impact critical load of structural instability under the critical condition of static load was obtained, as shown in Fig. 12b. Finally, the impact load was applied in both the horizontal and axial directions, and the dynamic load condition of specimen fracture was obtained, as shown in Fig. 12c. Figure 12d shows the fracture diagram of coal seam under dynamic and static loads.

Figure 12a shows the load displacement time curve of the sliding instability process of the combination structure with a weak layer under static load. The initial axial static load was 7 t, and the horizontal static load was 7 t. It can be seen from the horizontal load and displacement time in the figure that with the increase in horizontal static load, the horizontal displacement of the intermediate coal rock specimen (hereafter referred to as coal seam) shows three stages: slight increase, slow increase and sharp increase. When the displacement of the coal seam increases sharply, the load decreases rapidly. At the initial stage of loading, due to the axial load, there is a large cohesion in the weak plane, which restricts the slip of the coal seam. The displacement monitored by the measuring points at this stage was actually the deformation of the specimen when it was compressed, which showed that the displacement increases slightly; with the application of lateral load, the cohesion in the weak layer between rock and coal test blocks cannot completely restrict the slip of coal seam. The cohesion decreased gradually with the development of damage in the weak layer, and the displacement increases slowly; with the further increase in horizontal load, the weak layer was destroyed, and the cohesion in the weak layer disappears completely. Under the action of horizontal loading, the coal seam were unstable and sliding, which showed a sharp increase in displacement.

Figure 12b shows the load displacement time curve of the coal structure with a weak layer under horizontal dynamic load impact. It can be seen from the figure that the process time of coal seam sliding instability under horizontal dynamic loading was greatly shortened compared with that under static loading. The process from applying dynamic load impact to coal seam sliding instability lasts only 0.005 s and showed significant dynamic fracture characteristics. 0 ~ 0.0075 s in the figure was the static load stability stage. The 5 t dynamic load impact was preset at the end of the stability stage, but the load monitoring data show that the horizontal load was only increased by 3 t, and its displacement increases to 18 mm, which showed the fracture characteristics of rapid overall dumping.

Figure 12c shows the load and displacement curve with time of the sliding instability process of a rock coal structure with a weak layer under a two-way dynamic load. From the displacement time curve, the whole experimental process can be divided into the static load stability stage, deformation rebound stage, unstable equilibrium stage and unstable sliding stage. When the axial and horizontal impact loads were applied for the first time, there is a small deformation and rebound stage in the displacement in the horizontal directions, and then it remained stable for a period of time. Finally, under the action of static loading, the horizontal displacement increases rapidly, the coal seam slide, and the rock coal structure was unstable. From the unstable equilibrium stage to the sliding instability stage, the two-way static load remains stable, indicating that the instability of rock coal combination specimens was still caused by static load, but it is different from static load fracture or lateral dynamic load fracture.

Figure 12d shows the situation after the combined structure was damaged. It can be found that the whole coal starta slid toward the free face, yet there was an obvious main crack in the coal seam. The crack was 150 mm away from the free face, and the ratio to the height of the coal seam was 1.5, which showed that the sliding of coal seam was also accompanied by fracture.

#### Coal seam sliding fracture process and evolution characteristics of the displacement field

**Figure 13:**
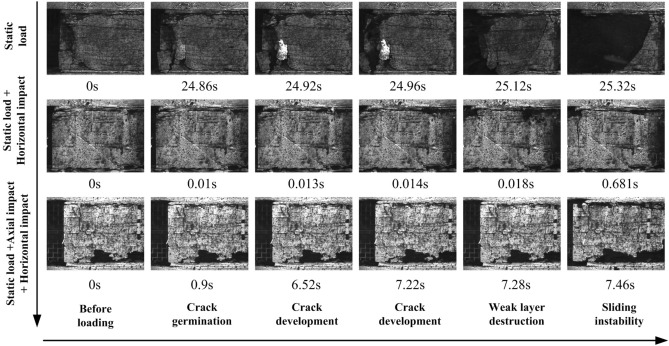
Coal seam sliding fracture process.

**Figure 14:**
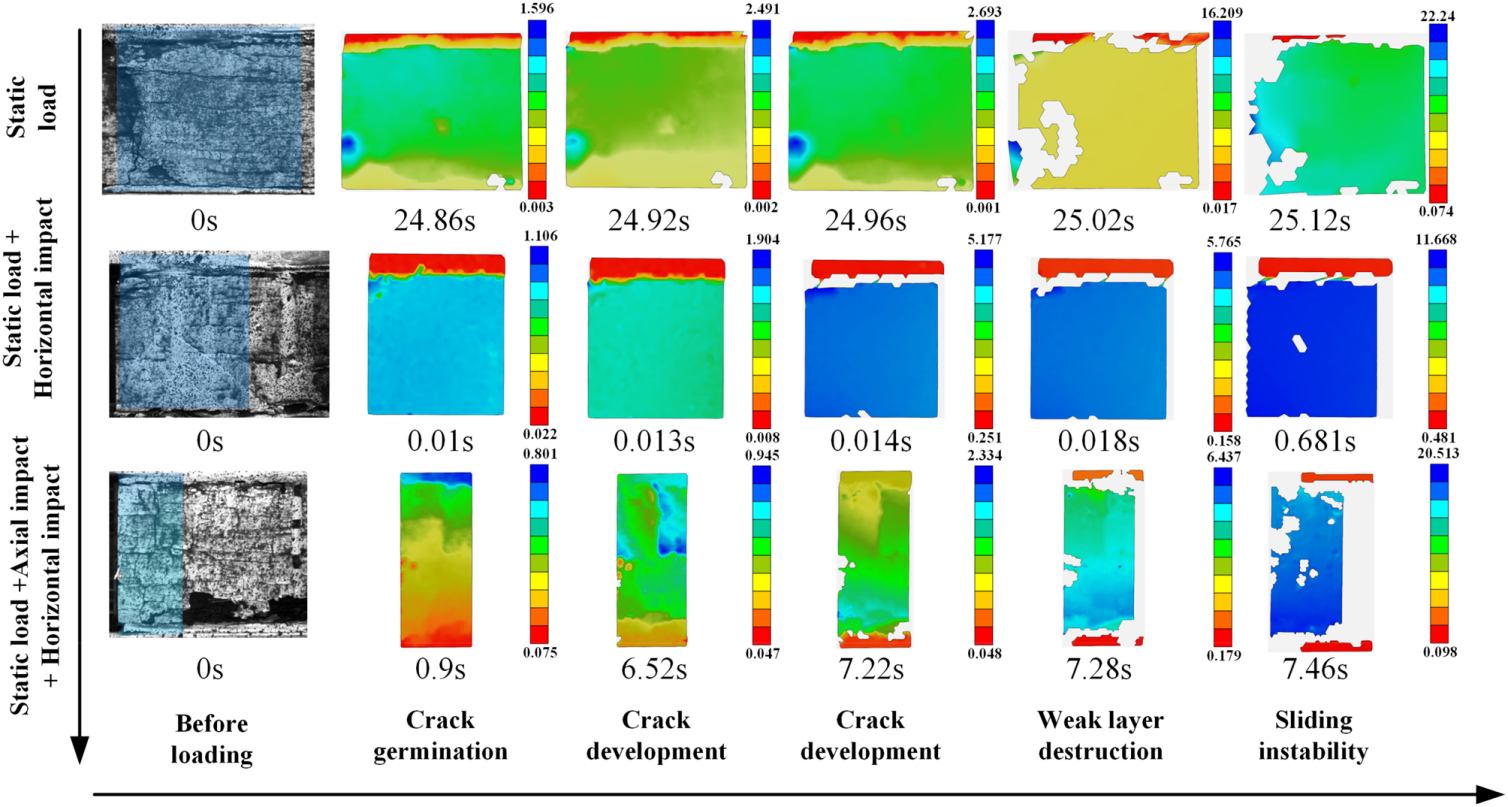
Evolution characteristics of the horizontal displacement field.

The whole fracture process of the rock coal structure was collected by a high-speed camera, as shown in Fig. 13. Additionally, the displacement field on the surface of the specimen was identified by DIC technology, as shown in Fig. 14.

Under the condition of static load, the coal seam slip was relatively slow. When the load reached 24.86 s, the coal blocks begin to fall off near the free face. With the continuous increase in horizontal load, a large number of fragments appear on the free face. From the cloud diagram of the displacement field shown in Fig. 13, it can also be found that the coal seam fracture starts from the free face, which was consistent with the results of the theoretical analysis and numerical simulation experiment. When the load reached 15.12 s, a large number of blocks began to fall off, and the coal seam slid as a whole. The whole process lasted approximately 0.26 s. It should be noted that the coal falling off was only the side falling off, and the whole coal seam were still complete, as shown in Fig. 14.

After the horizontal impact dynamic load was applied, the instability characteristics of the coal seam change significantly. The coal seam were always intact, showing the characteristics of overall sliding without fragments falling off. However, the instability process is fast, much faster than the static load experiment. From the displacement field nephogram in Fig. 14, it can be found that the whole sliding process lasted only 0.05 s.

The specimens subjected to horizontal impact and axial impact simultaneously showed the common characteristics of the first two experimental results. First, the coal seam slip lasted approximately 0.06 s, which was significantly faster than the static load condition, and the coal seam near the free face were not damaged. This showed that the dynamic load in two directions leads to the fracture of weak layers in the rock stratum and coal seam. Under the continuous action of static loading, the coal seam slide and lose stability.

Based on the above analysis, the instability form of the combination structure was closely related to the load application mode. The instability speed of coal seam caused by static load was slow. The instability of coal seam caused by the horizontal dynamic load will lead to the rapid dumping of the whole coal seam, fast instability speed, short time and high integrity of the coal seam. The structural instability caused by the two-way dynamic load has hysteresis, and the dynamic load will not lead to the direct instability of the coal seam but will first lead to the fracture of the weak layer, and then the coal seam slide rapidly. Additionally, it showed significant dynamic fracture characteristics, and the structural instability was more difficult to predict.

## Discussion

**Figure 15:**
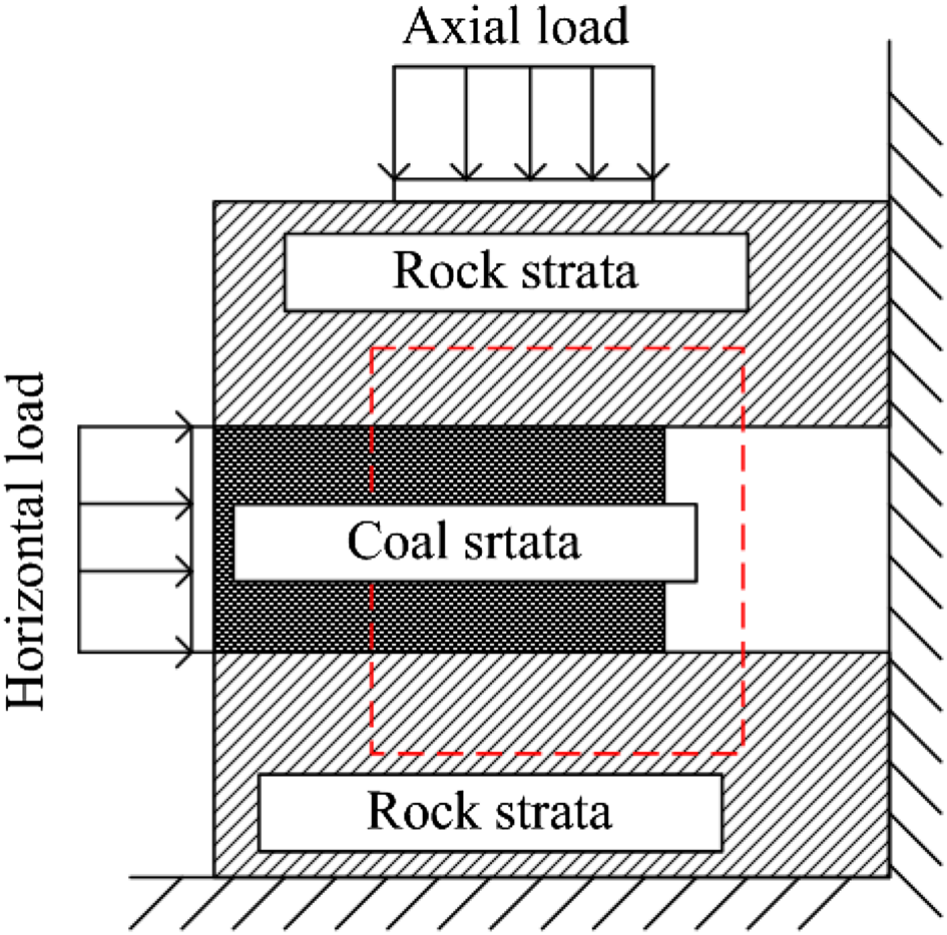
Stress condition of the test specimen.

In section "Experimental study on the sliding instability of a rock-coal combination structure with a weak layer under dynamic and static loads", combined coal rock structure sliding experiments under three load conditions were carried out to verify the weak layer sliding criterion and coal seam fracture criterion, and the verification results of the criterion in the experiment are further discussed. The loading mode of the experimental load is shown in Fig. 15.

**Figure 16:**
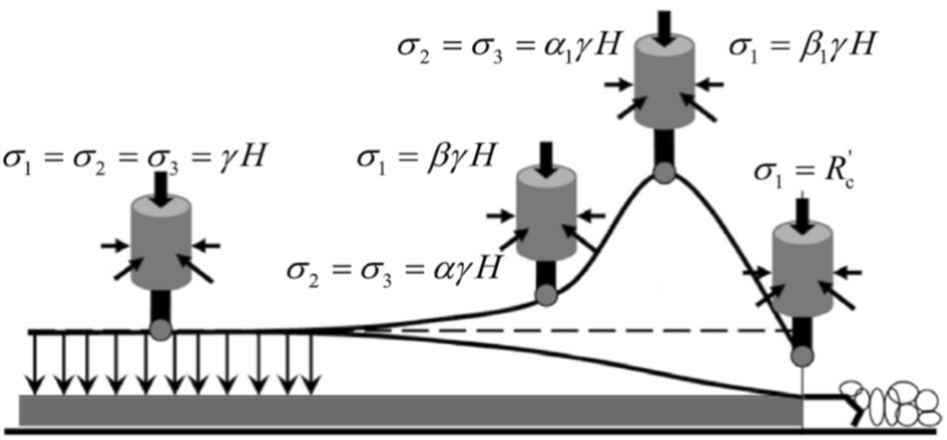
Stress state of coal and rock in front of the working face (Xie et al. 2011).

**Figure 17:**
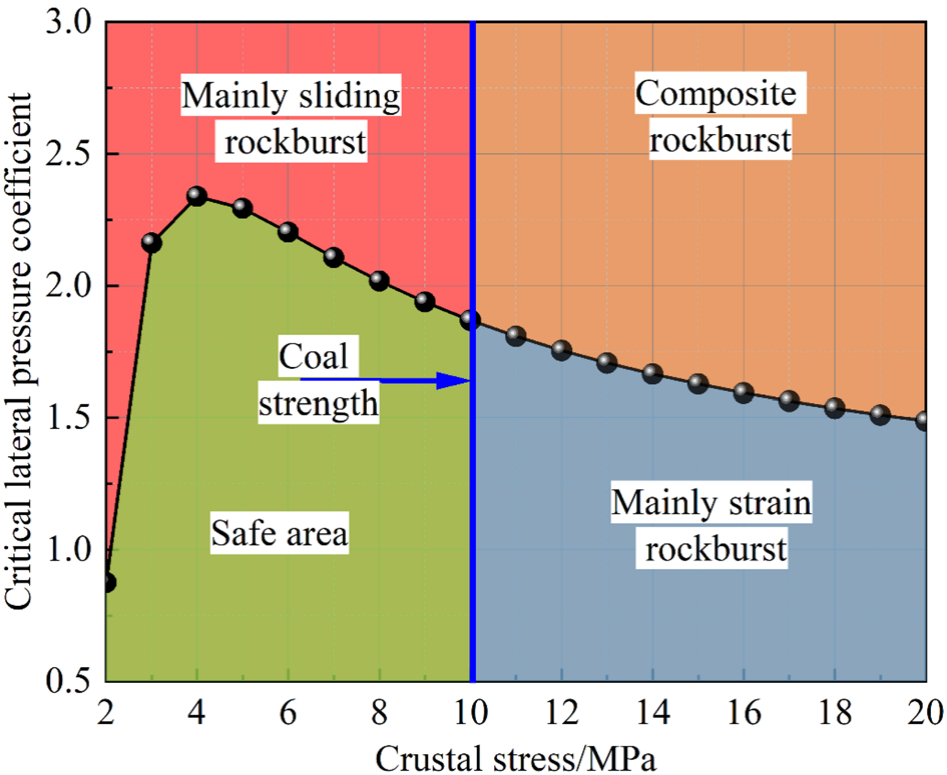
The relationship between the in situ stress and the coal seam slip critical lateral pressure coefficient.

The axial load was applied to a rigid indenter with a size of 100 × 100 mm. As coal is a kind of soft rock, this load application method may lead to stress concentration directly below the indenter, while the load at the position far away from the indenter may be unevenly distributed. The stress calculation of coal seam should be subject to the size of the indenter. In addition, the coal seam were cut into standard specimens and subjected to uniaxial compression testing. The average uniaxial compressive strength of coal was 8 MPa. Taking the mechanical parameters of the weak layer and the strength parameters of coal into the weak layer slip criterion and coal seam fracture criterion given in section "Slip fracture criterion of a combined coal and rock structure containing a weak layer", the critical lateral pressure coefficient of weak layer slip can be calculated as $K_{\lambda }^{W} { = }2.098$. It can be obtained that the lateral pressure coefficient during sliding was 2, close to $K_{\lambda }^{W}$, so the coal seam were not damaged but overall sliding. The damage to the coal seam under static loading may be caused by the deterioration of the mechanical properties of the coal sample surface. Overall, the experimental results are consistent with the physical experimental results. It can be seen that the $K_{\lambda }^{W}$ and $K_{{\sigma_{z} }}$ proposed in this study had a certain significance. Therefore, it is necessary to discuss the relationship between the manifestation of rockburst disasters and the judgment coefficient. Figure 16 shows the stress state of the coal and rock in front of the working face. Figure 17 shows the relationship between the in situ stress and the coal seam slip critical lateral pressure coefficient.

As the burial depth of the coal seam increases, the in situ stress also increases. To avoid the static load damage of coal seam caused by mining or excavation, it is often necessary to transfer the in situ stress of near-field surrounding rock to the deep part of coal seam. Therefore, the stress distribution characteristics of the working face shown in Fig. 16 are formed, and the stress distribution of the surrounding rock of the roadway was also similar. A complex geological structure and mining technology may lead to the redistribution of coal seam stress. For example, the subsidence of the goaf roof causes horizontal compression, which increased the horizontal in situ stress and reduced the in situ stress of the near-field surrounding rock. This leads to the uneven distribution of the lateral pressure coefficient in the surrounding rock of the roadway. The lateral pressure coefficient in some locations exceeds the safety value, and the in situ stress concentration in some locations exceeds the strength of the coal seam. Under the influence of the above conditions, the disturbance of the dynamic load easily induces compression fracture or overall sliding of the coal seam. It was characterized by strain rockburst and slip rockburst, or it has the common manifestation of two types of rockburst. Figure 17 shows the relationship between the in situ stress and the critical lateral pressure coefficient. The figure shows the critical in situ stress of coal seam fracture (10 MPa), which was calculated according to the mechanical parameters of the coal seam. It can be found that there will be no weak layer slip fracture under the lateral pressure coefficient curve; in contrast, it will induce sliding rockburst. When the local stress exceeds the critical value, it will exhibit strain rockburst or combined rockburst. For thick coal seam or thin coal seam roadway, the weak layer may invisibe, then the coal seam (thick coal pillar) provides the shear stress. And the lateral pressure coefficient of coal seam slip can be obtained by bringing the mechanical parameters of coal into the determination formula. The condition of thin coal seam road is more complicated. Therefore the rockburst type may be composite or strain, the critical lateral pressure coefficient determination method described may not be applicable to the thin coal seam roadway. The equipment will be further improved, and the acoustic emission and charge monitoring system will be supplemented to realize the identification of coal seam damage precursor information to achieve the purpose of identifying the type of rockburst and monitoring and early warning.

## Conclusions

This study focused on the slip damage mechanism of a coal rock combination structure containing a weak layer. First, it theoretically analyzed the stress state of a coal rock combination structure containing a weak layer, further proposes the criterion of slip damage of the weak layer and damage of coal, and then calibrates and verifies the criterion through numerical simulation. Finally, a sliding test of a rock coal structure with a weak layer under static and dynamic loads was carried out, and the instability law of the rock coal composite structure is analyzed, which was verified with theoretical analysis. The main conclusions were as follows.

1. The effect of the weak layer on maintaining roadway stability was theoretically analyzed, and the coal seam sliding lateral pressure coefficient caused by weak layer fracture and the theoretical value of overburden stress caused by coal seam fracture were obtained. The unknown parameters were calibrated and verified by numerical simulation. The key parameter p value of the near-field dangerous area of the roadway was closely related to the mechanical properties of the weak layer. This value has a power function relationship with the friction angle of the weak layer, a linear correlation with the cohesion of the weak layer, and a quadratic function relationship with the overburden stress. After fitting, the *P value* has a linear correlation with $K_{{\sigma_{z} }}$ and was further applied to determine the critical lateral pressure coefficient.
2. The sliding instability conditions and fracture forms of rock coal composite structures with weak layers under static load conditions, horizontal impact conditions and two-way impact conditions are studied. With the increase in horizontal load, the coal seam slides slowly. Before sliding and instability, a large number of fragments fell off, and the sliding process lasted for 0.26 s. Under the action of horizontal impact, the coal seam slip was rapid. The slip process lasted only 0.05 s. Under the action of a two-way impact load, the rock coal composite structure first deforms and rebounds, then remains stable, and finally slides and loses stability under the action of a static load. The fracture speed was between the first two load conditions and has a period of stability time, so the fracture was difficult to predict.
3. In this study, a determination method of the lateral pressure coefficient describing the sliding instability of a rock coal composite structure with a weak layer was proposed. The relationship between the lateral pressure coefficient and overburden stress was a power function. Combined with the critical overburden stress of coal seam fracture, the conversion mechanism between sliding rockburst and strain rockburst was discussed. According to the distribution characteristics of the stress field of the surrounding rock of the working face or roadway, the stress range of rockburst types was divided into safe area, strain rockburst, sliding rockburst and composite rockburst.

## Data Availability

The datasets used and/or analysed during the current study available from the corresponding author on reasonable request.
